# WORKPLACE SUPPORTS FOR WORKING LONGER: A CASE FOR THE BLACK LOW-WAGE WORKER

**DOI:** 10.1093/geroni/igz038.695

**Published:** 2019-11-08

**Authors:** Kendra Jason

**Affiliations:** University of North Carolina at Charlotte, Charlotte, North Carolina, United States

## Abstract

Government-sponsored retirement programs and employer benefits are directly tied to individual employment history and wages. Consequently, Black workers disproportionally face challenges in labor market compensation and retirement benefits. Due to a history of racial discrimination and economic oppression, Black workers earn less income over the life course and are less likely to have insurance support in comparison to their white counterparts in older age. This leads Black adults to remain in the workforce longer for financial support and presents unique physical and psychosocial challenges balancing work obligations and family responsibilities. Further, Black adults also suffer from more chronic illnesses, poor health outcomes, and death at higher rates compared to nearly all other racial groups. Drawing on data derived from a workplace case-study with interviews from 15 low-wage Black workers aged 50+ years, with multiple chronic conditions in the Southern United States. My aims are to (1) understand what workplace supports enable vulnerable workers to remain in the workforce, and (2) identity other buffers (i.e., resilience) to working with chronic conditions that enable prolonged work engagement. Findings suggest that workplaces can better support low-wage workers who cannot afford to retire by offering better pay and health benefits. Supervisor and coworker supports, flexible work arrangements and scheduling, and less stressful work environments also enable sustained work engagement. Research, policy and practice implications of this research include identifying workplace attributes and determining strategies to strengthen them, which is paramount to addressing disparities in work and health outcomes in the vulnerable communities.

